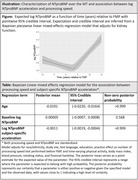# N‐terminal Brain Natriuretic Peptide over the Menopause Transition and Cognitive Performance after Menopause: Study of Women’s Heath Across the Nation

**DOI:** 10.1002/alz.086156

**Published:** 2025-01-09

**Authors:** Samar R. El Khoudary, James Matuk, Carol A. Derby, Daniel McConnell, Rebecca C Thurston

**Affiliations:** ^1^ University of Pittsburgh, Pittsburgh, PA USA; ^2^ Department of Neurology, and Department of Epidemiology and Population Health, Albert Einstein College of Medicine, Bronx, NY USA; ^3^ University of Michigan, Ann Arbor, MI USA

## Abstract

**Background:**

N‐terminal brain natriuretic peptide (NTproBNP) is a marker of cardiac health and a strong predictor of mortality, incident cardiovascular disease (CVD), and sudden cardiac death in community populations. A link between the menopause transition (MT), sex hormones, and NTproBNP has been suggested, though, no studies have formally examined how NTproBNP changes over the MT. In addition of being a marker of cardiac health, studies suggest NTproBNP to be related to cognitive performance, yet those studies have not considered the MT. We aim to 1) characterize NTproBNP changes over the MT, and 2) assess if the identified changes over the MT are associated with changes in cognitive performance post menopause.

**Method:**

Midlife women with a known date of the final menstrual period (FMP) for whom NTproBNP was measured for up to 5 time points across the MT and cognitive performance (processing speed measured using Symbol Digit Modalities test) was assessed repeatedly after FMP were included. Bayesian piecewise linear mixed‐effects models were used to estimate an inflection point and subject‐specific acceleration in NTproBNP relative to the FMP. Bayesian linear mixed‐effects models were used to relate subject specific acceleration in NTproBNP over the MT with cognitive performance changes in the post menopause.

**Result:**

The study included 1218 women (age at first included cognitive performance measure 53.8(SD: 3.14) years; 43.8% White, 28.4% Black, 11.0% Chinese, 12.3% Japanese, 4.5% Hispanic). NTproBNP significantly accelerated 3.2 years post FMP (posterior 95% Credible interval: 1.4, 5.2 years); posterior mean (95% Credible Interval) of log NTproBNP acceleration 0.043(0.036, 0.051) (**Illustration‐Figure**). Increased log NTproBNP acceleration over the MT was associated with greater processing speed decline post menopause [posterior mean (95% Credible Interval): ‐0.001(‐0.002, ‐0.0004) in SD unit processing speed per one SD unit increase in log NTproBNP acceleration] in adjusted model **(Illustration‐Table)**.

**Conclusion:**

Findings suggest a link between cardiac health during the MT and cognitive performance after menopause. The MT is a sensitive period of women’s lives characterized by accelerated increase in NTproBNP that in turn associates with a greater decline in postmenopausal processing speed.